# Variations in HIV Prevention Coverage in Subpopulations of Australian Gay and Bisexual Men, 2017–2021: Implications for Reducing Inequities in the Combination Prevention Era

**DOI:** 10.1007/s10461-023-04172-3

**Published:** 2023-09-27

**Authors:** Martin Holt, Curtis Chan, Timothy R. Broady, James MacGibbon, Limin Mao, Anthony K. J. Smith, John Rule, Benjamin R. Bavinton

**Affiliations:** 1 Centre for Social Research in Health, UNSW Sydney, Sydney, NSW 2052 Australia; 2 The Kirby Institute, UNSW Sydney, Sydney, Australia; 3 National Association of People With HIV Australia, Sydney, Australia

**Keywords:** Australia, Behavioural surveillance, Combination prevention, Gay and bisexual men, HIV prevention coverage, Men who have sex with men

## Abstract

Using repeated behavioural surveillance data collected from gay and bisexual men (GBM) across Australia, we assessed trends in HIV prevention coverage (the level of ‘safe sex’ achieved in the population by the use of effective prevention methods, including condoms, pre-exposure prophylaxis [PrEP] and having an undetectable viral load). We stratified these trends by age, country of birth/recency of arrival, sexual identity, and the proportion of gay residents in the participant’s suburb. Among 25,865 participants with casual male partners, HIV prevention coverage increased from 69.8% in 2017 to 75.2% in 2021, lower than the UNAIDS target of 95%. Higher levels of coverage were achieved among older GBM (≥ 45 years), non-recently-arrived migrants, and in suburbs with ≥ 10% gay residents. The lowest levels of prevention coverage (and highest levels of HIV risk) were recorded among younger GBM (< 25 years) and bisexual and other-identified participants. Younger, recently-arrived, and bisexual GBM were the most likely to use condoms, while PrEP use was concentrated among gay men, 25–44-year-olds, and in suburbs with more gay residents. The use of undetectable viral load was most common among participants aged ≥ 45 years. Our analysis shows that high HIV prevention coverage can be achieved through a mixture of condom use, PrEP use, and undetectable viral load, or by emphasising PrEP use. In the Australian context, younger, bisexual and other-identified GBM should be prioritised for enhanced access to effective HIV prevention methods. We encourage other jurisdictions to assess the level of coverage achieved by combination prevention, and variations in uptake.

**Supplementary Information:**

The online version contains supplementary material available at 10.1007/s10461-023-04172-3.

## Resumen

Utilizando datos repetidos de vigilancia conductual recopilados de hombres homosexuales y bisexuales (GBM) en toda Australia, evaluamos las tendencias en la cobertura de la prevención del VIH (el nivel de "sexo seguro" logrado en la población mediante el uso de métodos de prevención eficaces, incluyendo condones, Profilaxis de Pre-Exposición al VIH [PrEP] y tener una carga viral indetectable). Estratificamos estas tendencias por edad, país de nacimiento/tiempo desde la llegada al país, identidad sexual y proporción de residentes homosexuales en el suburbio del participante. Entre 25.865 participantes con parejas masculinas ocasionales, la cobertura de prevención del VIH aumentó del 69,8% en 2017 al 75,2% en 2021, cifra inferior al objetivo de ONUSIDA del 95%. Se lograron niveles más altos de cobertura entre GBM de mayor edad (≥45 años), inmigrantes no llegados recientemente y en suburbios con ≥10% de residentes homosexuales. Los niveles más bajos de cobertura de prevención (y los niveles más altos de riesgo de VIH) se registraron entre los GBM más jóvenes (<25 años) y los participantes bisexuales y con otras identidades. Los GBM más jóvenes, recién llegados y bisexuales fueron los más propensos a usar condones, mientras que el uso de PrEP se concentró entre hombres homosexuales, de 25 a 44 años, y en los suburbios con más residentes homosexuales. El uso de carga viral indetectable fue más común entre los participantes de ≥45 años. Nuestro análisis demuestra que se puede lograr una alta cobertura de prevención del VIH mediante una combinación del uso de condón, uso de PrEP y carga viral indetectable, o enfatizando el uso de PrEP. En el contexto australiano, se debe dar prioridad a los GBM más jóvenes, bisexuales y con otras identidades para mejorar el acceso a métodos eficaces de prevención del VIH. Alentamos a otras jurisdicciones a evaluar el nivel de cobertura logrado mediante la prevención combinada y las variaciones en la adopción.

## Introduction

In the 2010s, the discovery that HIV pre-exposure prophylaxis (PrEP) and treatment as prevention (TasP) were highly effective prevention strategies ushered in a new era of ‘biomedical HIV prevention’ [1–6]. PrEP and TasP have expanded the options for a ‘combination prevention’ approach, in which a range of effective strategies are made available and promoted to people at risk of HIV [7, 8]. The combination prevention approach was advanced based on the recognition that multiple prevention options may be required to achieve effective prevention coverage and reduce HIV incidence [9, 10]. The approach also acknowledges that the acceptability of different prevention options varies within and between populations, and that strategies should be tailored to local HIV epidemics and affected communities.

The expansion of the range of effective HIV prevention strategies has created hope that HIV infection rates can be reduced, and that HIV epidemics can be slowed or stopped. However, the development of new prevention approaches does not necessarily overcome inequities in access to and the effective use of HIV prevention [11]. Combination prevention also raises technical challenges in monitoring and evaluation, such as understanding the differential uptake of methods in different subpopulations and places, and the overall level of prevention coverage achieved when a range of methods are being used [12, 13]. It is unclear whether access to combination methods reduces or exacerbates known inequities in prevention. Here we attempt to address these issues with reference to a specific HIV-affected population: gay and bisexual men (GBM) in Australia. Our aim is to show that it is possible to assess prevention coverage and the range of strategies by key demographic characteristics, illuminating variations in uptake, preferences for different strategies and opportunities to increase coverage.

Researchers have previously noted that longstanding measures of HIV risk and safety (such as engaging in condomless sex or consistent condom use) are insufficient to properly assess HIV risk and protection in populations where there is substantial use of PrEP and TasP [14, 15]. Intercourse may not be ‘protected’ by barrier methods like condoms but it may instead be protected by biomedical HIV prevention methods [16]. We have previously developed a way to measure the use of different strategies (including condoms, PrEP and TasP) and the overall level of HIV prevention coverage among GBM in Australia who have casual sex [13, 15, 17]. This is similar to reporting from other settings which have embraced biomedical prevention among GBM [18, 19]. However, in general we note there has been an absence of analysis of changes in the range of prevention strategies used by GBM as PrEP and TasP become more commonly used, coincident with a lack of consistency of investment in routine behavioural surveillance with key populations [20, 21]. Some studies still report changes in the use of strategies like condom use or PrEP in isolation [22], which may underestimate the level of prevention coverage in the population and the range of strategies used, and ignores how the introduction of a new strategy (e.g. PrEP) may affect existing strategies (e.g. condoms) [23, 24]. Our work shows that since PrEP was introduced in Australia, condom use has fallen, viral suppression has remained high among GBM living with HIV, and PrEP use by HIV-negative men has rapidly increased, resulting in a change in the mix of prevention strategies used and an overall increase in prevention coverage [13, 17]. These changes appear to have led to the first substantial declines in annual HIV diagnoses among GBM in Australia for 15 years [25, 26].

An overall increase in HIV prevention coverage due to the adoption of a combination prevention approach may disguise variability in access to and the use of different prevention methods by subpopulations [10, 27]. The uptake of PrEP, for example, has been faster and achieved higher levels of coverage among older, white gay men in some metropolitan areas of Australia, central Asia, Europe and the United States [27–32]. As biomedical HIV prevention has become more commonly used in Australia, studies have found that younger GBM, bisexual men and recently-arrived Asian-born men report less access to and use of PrEP, and higher levels of HIV risk [13, 28, 33–36]. In 2021, 19% of Australia’s HIV diagnoses were reported among Asian-born GBM, despite only 13% of Australia’s population being born in Asia [26, 37]. (In 2021, 29% of Australia’s population was born overseas.) Some HIV-related disparities in Australia are related to access to healthcare. Australia has a subsidised health system which means that permanent residents and citizens can attend a doctor for free and pay a maximum of AU$30 for prescriptions. In practice, many general practitioners charge a fee for consultations and most temporary residents are ineligible for subsidised medicines, which may make accessing PrEP (or HIV treatment) unaffordable [33, 38]. COVID-19 appears to have exacerbated some HIV-related disparities in Australia, with younger GBM, bisexual men and GBM from suburbs with fewer gay residents reporting greater reductions in partner numbers, HIV testing and PrEP use in response to COVID-19, but more risk of HIV, if they continued to have casual sex [39].

Despite the disruptions generated by COVID-19, Australia remains committed to HIV prevention targets, such as the virtual elimination of HIV transmission by 2025 [40, 41]. Globally, there are calls to increase HIV prevention coverage in key populations to 95%, using combination prevention methods to respond to inequities in coverage [10, 11]. Responding to this call, we used national behavioural surveillance data collected from GBM to assess HIV prevention coverage by key demographic characteristics, with the aim of better understanding the range of strategies used by different subgroups of GBM, and areas where coverage could be improved.

## Method

### Participants and Procedures

Data were collected through repeated, cross-sectional, behavioural surveillance surveys of GBM (the Gay Community Periodic Surveys) conducted during LGBTQ festival periods in seven of Australia’s states and territories [15, 39]. The surveys have been conducted since 1996, and occur annually in New South Wales, Queensland and Victoria, and every two years in the other jurisdictions. Traditionally, most recruitment is conducted in person by trained peers at gay venues and events, supplemented by online advertising and recruitment. During COVID-19 restrictions (particularly during 2020–2021), most recruitment was conducted online [39].

Eligible participants are residents of Australia who identify as male (including cisgender and transgender men), who are at least 16 years old (online participation) or 18 years old (in person recruitment), and who identify as gay, bisexual or queer and/or who have had sex with a man in the past 5 years. Participants are asked to complete a questionnaire containing demographic items, and questions about HIV and sexual health testing, HIV status, HIV treatment and viral load, use of different prevention methods including condoms and PrEP, sex with casual and regular male partners, relationships, and drug use. Recall periods for behaviour are typically 6 or 12 months. Participation is typically anonymous with no contact details collected, unless online participants opt to receive feedback on the survey, in which case contact details are stored separately to questionnaire responses.

For field-based, in person recruitment, trained peers approach potential participants at venues and events during scheduled shifts, and provide study information and the questionnaire to consenting participants. Participants fill in a paper copy of the questionnaire in English and return it to the recruiters. For online recruitment, potential participants are directed by advertising to the study website (https://gcpsonline.net), participant information and the questionnaire, hosted on Qualtrics (Provo, UT). Paid advertising is commonly used on Facebook, Instagram and Grindr. The online questionnaire has the same questions as the paper questionnaire but uses adaptive routing to exclude ineligible participants and hide irrelevant questions. The online questionnaire is available in English and seven other languages [39]. Completing the questionnaire is taken as evidence of consent. Participants who complete less than half the questionnaire are considered to have withdrawn and their responses are deleted. The study has institutional ethics approval from the UNSW Sydney Human Research Ethics Committee (HC180903) and the research review committees of the community organisations ACON and Thorne Harbour Health.

### Measures

The current survey measures have been previously described [15, 39]. The primary outcome measure used in these analyses was net HIV prevention coverage for participants who reported sex with casual male partners in the previous six months; in Australia, casual sex between men remains the primary transmission context for HIV [13, 15, 17, 42]. Net prevention coverage includes any safe sex strategy (avoiding anal intercourse, consistent condom use, PrEP or TasP), and is constructed from these mutually exclusive categories [13]:

1. No anal intercourse.
2. Consistent condom use.
3. HIV-positive, on HIV treatment, has an undetectable viral load and reports condomless anal intercourse with casual male partners (CAIC), indicating TasP.
4. HIV-negative, on PrEP, and reports CAIC.
5. HIV-positive, not on treatment or detectable viral load and reports CAIC.
6. HIV-negative or untested, not on PrEP, and reports CAIC.

Categories 1–4 are considered ‘safe sex’ and are summed to calculate net prevention coverage. Categories 5 and 6 represent sex with a risk of HIV transmission. Participants in Category 6 are regarded as at the highest risk of HIV infection. These categories are constructed from participant responses to questions about sex in the last six months with casual male partners, including whether anal intercourse occurred, and the frequency of condom use and condomless sex. The categories also require the participant’s answers to questions about self-reported HIV status, PrEP use, and if living with HIV, the use of HIV treatment at the time of the survey and latest viral load test result (‘Undetectable’, ‘Detectable’, or ‘Unsure/don’t know’).

To describe the sample and to conduct stratifications of net prevention coverage and the use of different prevention strategies, we included the demographic characteristics age, country of birth, sexual identity, proportion of gay residents in the participant’s suburb (based on a previously published method) [43], and state/territory of residence. To adjust for variations in sampling, we also included recruitment source.

### Analyses

All analyses were conducted using Stata version 14.2 (StataCorp LLC, College Station, TX, USA). We report descriptive statistics for the sample (participants who reported sex with casual male partners in the six months prior to survey) and trends in demographic characteristics and recruitment source for the period 2017–2021. This period was selected to include the year before PrEP became publicly subsidised in 2018, and then four years in which PrEP use increased. The trend in the mean age of the sample was assessed with linear regression, with year as the independent variable. Trends in categorical variables were assessed with logistic regression with year as the independent variable and the demographic variable or recruitment source as the dependent variable. Odds ratios (OR), 95% confidence intervals (CI) and *p* values are reported. Most of the dependent variables were dichotomised in the trend analyses, e.g. age (< 25 vs. ≥ 25 years), country of birth (Australia vs. overseas), sexual identity (gay vs. bisexual/other), proportion of gay residents (< 10% vs. ≥ 10%) and recruitment source (venue or event vs. online).

Prevalence and trends in net prevention coverage, the use of different prevention strategies and risk of HIV are reported nationally (for all states/territories combined). Trends were assessed with multivariate logistic regression with year as the independent variable and each of the six mutually exclusive categories described above as a dependent variable (with each category compared with all of other others in turn, e.g. No anal intercourse vs. the other categories). Demographic variables and recruitment source were included as covariates in these trend analyses, to control for variations in sampling. Adjusted odds ratios (AOR), 95% confidence intervals and *p* values are reported.

We stratified net prevention coverage, the use of different prevention strategies and HIV risk by the demographic variables noted above: age, country of birth, sexual identity, and proportion of gay residents. Age was grouped into these categories: < 25 years, 25–44 years, ≥ 45 years. Country of birth and sexual identity were categorised to highlight potential targets for HIV prevention e.g. Australian-born vs. recently-arrived migrants vs. non-recently-arrived migrants; gay-identified participants vs. bisexual/other-identified participants. Trends in net prevention coverage, the use of different prevention strategies and risk of HIV were assessed with multivariate logistic regression, with demographic variables and recruitment source included as covariates.

## Results

During 2017–2021, a total of 42,772 survey responses were collected. Of these, 25,865 were from participants who reported sex with casual male partners in the six months prior to survey. Of these 25,865 participants, 25,107 (97.1%) were cisgender and 298 (1.2%) transgender men (and 560 reported other gender identities), the mean age was 37.6 years (standard deviation = 13.0), and 18,055 (69.8%) were born in Australia and 7,821 (30.2%) overseas. Most participants identified as gay (n = 22,821, 88.2%), with 2,269 (8.8%) participants identifying as bisexual, 85 (0.3%) as heterosexual and 705 (2.7%) another identity. A minority of the sample was educated up to high school level (n = 5841, 22.6%), 5386 (20.9%) had a trade certificate, and 14,563 (56.5%) had a university degree. Nearly two-thirds (65.0%) of the sample was employed full-time (n = 16,806), 3524 (13.6%) part-time, and 2314 (8.9%) were students. Most participants (n = 20,775, 80.9%) lived in a suburb with < 10% gay residents and 4908 (19.1%) lived in a suburb with ≥ 10% gay residents. Two-thirds of responses (n = 17,755, 68.4%) were from participants recruited in person at venues and events, while 8210 (31.6%) were recruited online. Participants were most likely to reside in New South Wales (n = 9151, 35.2%), Queensland (n = 4712, 18.2%) and Victoria (n = 8857, 34.1%), with the remainder (n = 3245, 12.5%) from the other states and territories. Most participants reported that they were HIV-negative (n = 21,542, 84.4%), with 2462 (9.7%) indicating they were living with HIV and 1522 (6.0%) were untested (never tested for HIV) or did not know their HIV status (tested but had not received or returned for a result). Among HIV-negative and untested/unknown status participants during 2017–2021 (n = 21,373), 8483 (39.7%) reported that they had used PrEP in the six months prior to survey. Of these 8483 PrEP users, 98.1% (n = 8322) indicated that they were HIV-negative and the remainder either did not answer the question about HIV status (n = 73, 0.9%) or said they did not know their HIV status (n = 88, 1.0%). Among participants living with HIV who answered questions on both HIV treatment and viral load (n = 2311), 2,186 (94.6%) were on HIV treatment at the time of the survey and 2180 (94.3%) indicated that their last HIV viral load test was undetectable.

Overseas-born participants were only asked to specify their country of birth and length of residence in Australia in the questionnaire from 2019 onwards. Of those born overseas who participated during 2019–2021 (n = 4456), the most common countries of birth were the United Kingdom (n = 688), New Zealand (n = 493), United States (n = 197), Malaysia (n = 192), Philippines (n = 188), South Africa (n = 140), China (n = 133), Brazil (n = 125), India (n = 121) and Colombia (n = 101). Of those born overseas who participated during 2019–2021, 674 (15.1%) had been resident in Australia in for less than 2 years (recently-arrived migrants) and 3782 (84.9%) had been resident in Australia for two or more years (non-recently-arrived).

Table 1 shows trends in participants’ demographic characteristics, recruitment source and self-reported HIV status. The mean age of the sample increased from 36.1 to 40.6 years during 2017–2021, with most of the increase occurring in 2020–2021 (F = 243.50 [df = 1,25852], β = 0.92, *p* < 0.001). That meant that the proportion of participants aged < 25 years declined between 2017 and 2021. The proportions of participants born in Australia and overseas remained stable, but the proportion of gay-identified participants declined and the proportions of bisexual and other-identified participants increased (with the largest change in 2021, coincident with the increase in online recruitment during COVID-19). The proportion of participants from postcodes with ≥ 10% gay residents declined over time (with the largest change between 2020 and 2021). The proportion of participants recruited online increased over time, with a large increase in 2021 as COVID-19 interrupted face-to-face recruitment [39]. The proportion of participants from New South Wales remained stable over time, decreased from Queensland, and increased from Victoria and the other jurisdictions. Compared with HIV-negative participants in the sample, the proportion of untested/unknown status participants increased over time, while the proportion of HIV-positive participants remained stable. In the analyses of trends in HIV prevention coverage that follow, age, sexual identity, proportion of gay residents, recruitment source and state/territory are included as covariates to control for variations in sampling if they were not already included as an independent variable (e.g. to create a stratification).

**Table 1:** Trends in participants’ demographic characteristics, recruitment source and self-reported HIV status

	2017 n (%)	2018 n (%)	2019 n (%)	2020 n (%)	2021 n (%)	OR (95% CI)	*p* value
Age							
< 25	954 (16.3)	817 (15.3)	835 (14.6)	619 (12.3)	461 (11.6)	Ref 1.11	Ref < 0.001
≥ 25	4909 (83.7)	4539 (84.7)	4899 (85.4)	4411 (87.7)	3521 (88.4)	(1.08–1.14)	
Country of birth							
Australia	4107 (70.5)	3744 (70.4)	3888 (68.7)	3435 (68.7)	2810 (70.7)	Ref	Ref
Overseas	1717 (29.5)	1572 (29.6)	1771 (31.3)	1568 (31.3)	1164 (29.3)	1.01 (0.99–1.03)	0.310
Sexual identity^a^							
Gay	5310 (91.1)	4773 (89.6)	5042 (89.2)	4348 (87.1)	3257 (81.9)	0.83 (0.81–0.86)	< 0.001
Bisexual	373 (6.4)	403 (7.6)	431 (7.6)	476 (9.5)	582 (14.6)	1.24 (1.20–1.28)	< 0.001
Heterosexual/other	145 (2.5)	153 (2.9)	181 (3.2)	170 (3.4)	137 (3.5)	1.09 (1.04–1.15)	0.001
Proportion of gay residents in participant’s suburb							
< 10%	4691 (80.6)	4289 (80.6)	4484 (80.3)	3775 (76.2)	3468 (88.8)	Ref	Ref
≥ 10%	1129 (19.4)	1035 (19.4)	1099 (19.7)	1179 (23.8)	436 (11.2)	0.94 (0.92–0.96)	< 0.001
Recruitment location							
Venue or event	4930 (84.2)	4107 (76.8)	4428 (78.1)	3465 (69.1)	378 (9.5)	Ref	Ref
Online	923 (15.8)	1238 (23.2)	1241 (21.9)	1552 (30.9)	3603 (90.5)	2.07 (2.03–2.12)	< 0.001
State/territory^a^							
New South Wales	2099 (35.9)	1820 (34.1)	1916 (33.8)	2045 (40.8)	1226 (30.8)	0.99 (0.97–1.01)	0.409
Victoria	1912 (32.7)	1744 (32.6)	1878 (33.1)	1911 (38.1)	1368 (34.4)	1.04 (1.02–1.06)	< 0.001
Queensland	1260 (21.5)	1132 (21.2)	1090 (19.2)	583 (11.6)	642 (16.1)	0.87 (0.85–0.89)	< 0.001
Other	582 (9.9)	649 (12.1)	785 (13.9)	478 (9.5)	745 (18.7)	1.13 (1.10–1.16)	< 0.001
Self-reported HIV status							
HIV-negative	4754 (84.5)	4416 (84.7)	4861 (85.9)	4232 (85.6)	3279 (82.4)	Ref	Ref
HIV-positive	565 (10.0)	526 (10.1)	482 (8.5)	393 (8.0)	396 (10.0)	0.97 (0.94–1.00)	0.073
Untested/unknown	310 (5.5)	271 (5.2)	316 (5.6)	320 (6.5)	305 (7.7)	1.09 (1.05–1.14)	< 0.001
Total	5853	5345	5669	5017	3981	25,865	

*OR* odds ratio, *CI* confidence interval

^a^Each category compared with all the others for trend tests

### Trends in HIV Prevention Coverage

**Fig. 1:**
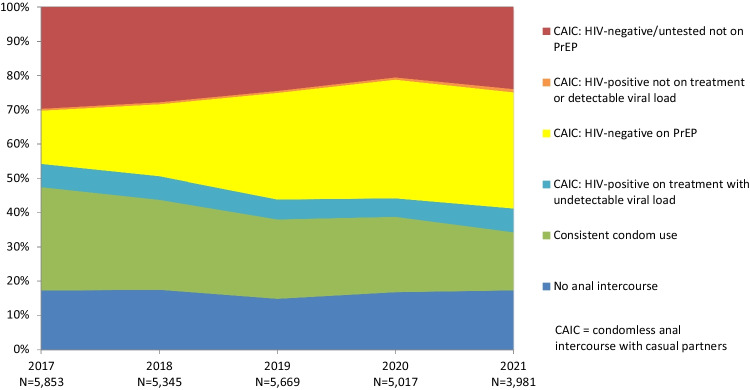
Trends in the use of different prevention strategies and HIV risk among participants with casual male partners in the six months prior to survey

Table 2 and Fig. 1 shows national trends in net HIV prevention coverage and the use of different prevention strategies for participants with casual male partners. The proportion of participants reporting no anal intercourse with casual partners remained stable during 2017–2021, while the proportion reporting consistent condom use fell. The proportion of participants reporting any condomless sex with casual partners increased markedly. The proportion of participants who were HIV-positive, had an undetectable viral load and reported condomless sex decreased, while the proportion of participants on PrEP who reported condomless sex increased markedly. The proportion of participants who were HIV-positive, not on treatment or had a detectable load and reported condomless sex remained low and stable (under 1%), while the proportion of participants who were at higher risk of HIV infection (HIV-negative or untested, not on PrEP, who reported condomless sex) declined over time. HIV prevention coverage increased over time, peaking at 78.7% in 2020.

**Table 2:** Trends in national HIV prevention coverage, the use of different prevention strategies and HIV risk among participants with casual male partners in the six months prior to survey

	2017 n (%)	2018 n (%)	2019 n (%)	2020 n (%)	2021 n (%)	AOR (95% CI)	*p* value
No anal intercourse	1008 (17.2)	933 (17.5)	840 (14.8)	840 (16.7)	688 (17.3)	0.97 (0.95–1.00)	0.054
Consistent condom use	1771 (30.3)	1406 (26.3)	1315 (23.2)	1106 (22.0)	676 (17.0)	0.85 (0.83–0.87)	< 0.001
Any condomless anal intercourse	3074 (52.5)	3006 (56.2)	3514 (62.0)	3071 (61.3)	2617 (65.8)	1.15 (1.12–1.17)	< 0.001
*Subcategories of participants who had condomless anal intercourse*							
HIV-positive on treatment with undetectable viral load	393 (6.7)	365 (6.8)	331 (5.8)	271 (5.4)	277 (7.0)	0.93 (0.90–0.97)	0.002
HIV-negative on PrEP	911 (15.6)	1125 (21.0)	1763 (31.1)	1737 (34.6)	1349 (33.9)	1.43 (1.40–1.47)	< 0.001
HIV-positive not on treatment or detectable viral load	32 (0.5)	31 (0.6)	32 (0.6)	33 (0.7)	38 (1.0)	0.98 (0.86–1.11)	0.753
HIV-negative/untested not on PrEP	1738 (29.7)	1485 (27.8)	1388 (24.5)	1030 (20.6)	953 (23.9)	0.86 (0.84–0.88)	< 0.001
Net prevention coverage	4083 (69.8)	3829 (71.6)	4249 (74.9)	3954 (78.7)	2990 (75.2)	1.17 (1.14–1.19)	< 0.001
Total	5853	5345	5669	5017	3981		

*AOR* adjusted odds ratio, *CI* confidence interval

### Variations in Prevention Coverage by Age

**Fig. 2:**
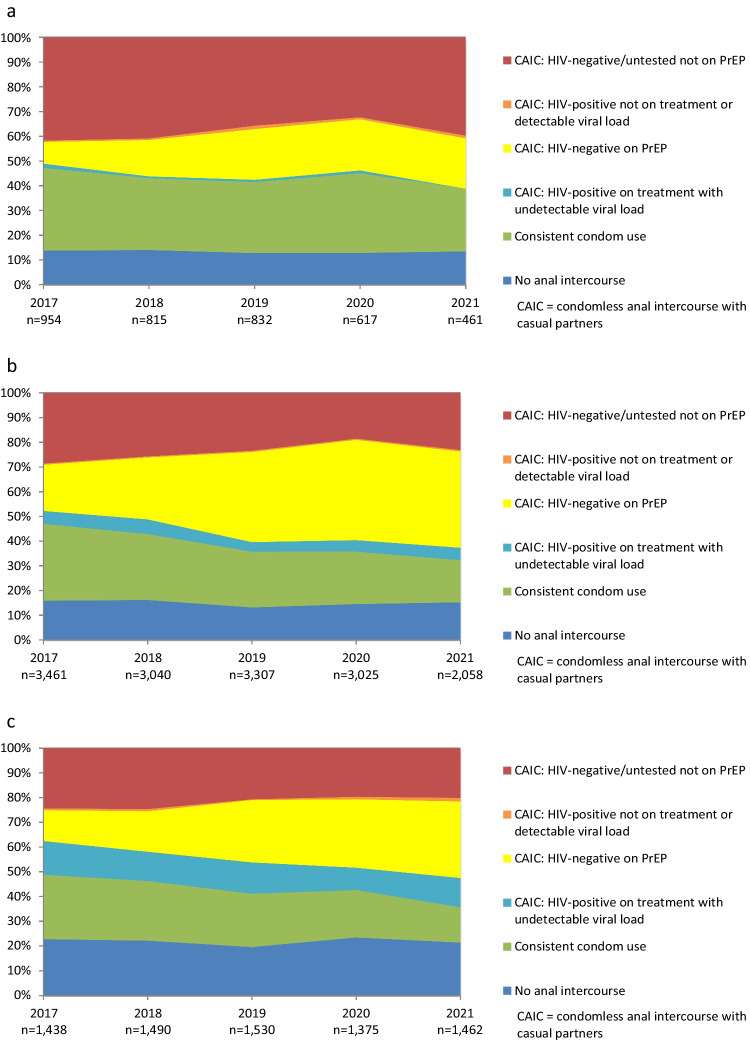
**a** Trends in the use of different prevention strategies and HIV risk with casual male partners among participants aged < 25 years. **b** Trends in the use of different prevention strategies and HIV risk with casual male partners among participants aged 25–44 years. **c** Trends in the use of different prevention strategies and HIV risk with casual male partners among participants aged ≥ 45 years

Figure 2a–c show trends in HIV prevention coverage and the use of different prevention strategies, stratified by age group (see also Supplemental Tables 1–3). Out of the three age groups, the youngest participants (< 25 years; Fig. 2a, Supp Table 1) consistently had the lowest level of prevention coverage, although coverage improved slightly over time from 57.8% in 2017 to 59.2% in 2021 (AOR = 1.10, 95% CI 1.04–1.16, *p* = 0.001). Participants aged < 25 years were consistently the least likely to report no anal intercourse with casual partners (from 13.7% in 2017 to 13.4% in 2021; AOR = 0.99, 95% CI 0.92–1.07, *p* = 0.80), i.e. the youngest participants were the most likely to report anal sex with casual partners. Participants aged < 25 years were also the most likely to report consistent condom use with casual partners, although this decreased from 33.5% in 2017 to 25.4% in 2021 (AOR = 0.93, 95% CI 0.88–0.98, *p* = 0.01). Participants aged < 25 years were the least likely to use PrEP when having condomless sex with casual partners, although use increased from 8.8% in 2017 to 20.4% in 2021 (AOR = 1.37, 95% CI 1.27–1.47, *p* < 0.001). The < 25 age group had very few participants living with HIV, compared with the older age groups. Participants aged < 25 years consistently had the highest proportion of participants at higher risk of HIV infection, although this decreased slightly from 41.7% in 2017 to 39.7% in 2021 (AOR = 0.91, 95% CI 0.86–0.96, *p* < 0.001).

Prevention coverage among participants aged 25–44 years (Fig. 2b, Supp Table 2) increased from 70.9% in 2017 to 76.3% in 2021 (AOR = 1.17, 95% CI 1.14–1.21, *p* < 0.001). The proportion of 25–44-year-olds reporting consistent condom use fell from 31.1% in 2017 to 17.0% in 2021 (AOR = 0.83, 95% CI 0.80–0.85, *p* < 0.001). Participants aged 25–44 years were the most likely to report PrEP use and condomless sex (compared with the other age groups), increasing from 18.7% in 2017 to 39.0% in 2021 (AOR = 1.42, 95% CI 1.38–1.47, *p* < 0.001). In the 25–44 age group, the proportion of participants who were HIV-positive, had an undetectable viral load and reported condomless sex declined slightly over time (from 5.2% in 2017 to 5.1% in 2021; AOR = 0.93, 95% CI 0.88–0.99, *p* = 0.03), while there were relatively few participants in this age group who were HIV-positive, detectable or not on treatment, and reported condomless sex (0.4–0.6%). The proportion of 25–44-year-olds who were at higher risk of HIV infection decreased from 28.6% in 2017 to 23.1% in 2021 (AOR = 0.85, 95% CI 0.83–0.88, *p* < 0.001).

The highest level of prevention coverage by age was observed in participants aged ≥ 45 years, increasing from 75.0% in 2017 to 78.5% in 2021 (AOR = 1.17, 95% CI 1.12–1.22, *p* < 0.001; Fig. 2c, Supp Table 3). Participants in this age group were consistently the most likely to report no anal intercourse with casual partners (from 22.7% in 2017 to 21.3% in 2021; AOR = 0.98, 95% CI 0.93–1.03, *p* = 0.36), while consistent condom use declined from 26.1% in 2017 to 14.3% in 2021 (AOR = 0.87, 95% CI 0.83–0.91, *p* < 0.001). PrEP use by those reporting condomless sex increased in this age group from 12.5% in 2017 to 31.0% in 2021 (AOR = 1.44, 95% CI 1.37–1.52, *p* < 0.001). Participants aged ≥ 45 years also featured the highest levels of HIV-positive participants with undetectable viral loads who reported condomless sex (from 13.6% in 2017 to 11.8% in 2021; AOR = 0.94, 95% CI 0.89–1.00, *p* = 0.051). There were relatively few participants in the ≥ 45 years age group who were HIV-positive, detectable or not on treatment, and reported condomless sex (0.3–1.4%). This age group had the smallest proportion of participants who were at higher risk of HIV infection, decreasing from 24.3% in 2017 to 20.1% in 2021 (AOR = 0.85, 95% CI 0.81–0.89, *p* < 0.001).

### Variations in Prevention Coverage by Country of Birth and Recency of Arrival

**Fig. 3:**
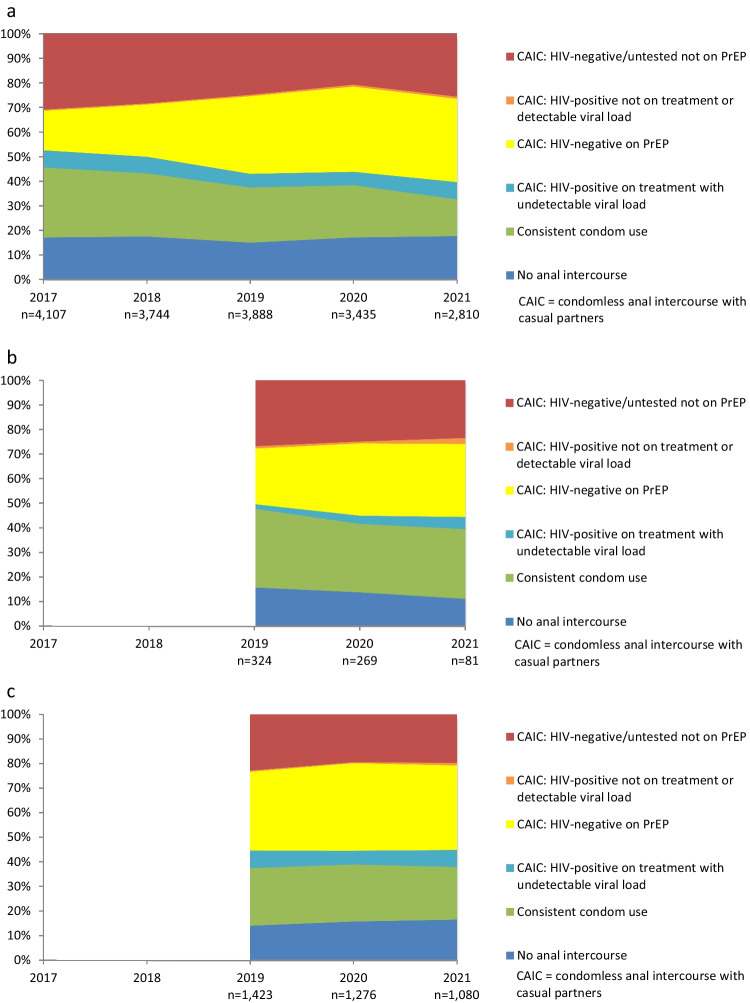
**a** Trends in the use of different prevention strategies and HIV risk with casual male partners among participants born in Australia. **b** Trends in the use of different prevention strategies and HIV risk with casual male partners among recently-arrived overseas-born participants. **c** Trends in the use of different prevention strategies and HIV risk with casual male partners among non-recently-arrived overseas-born participants

Figure 3a–c show trends in HIV prevention coverage and the use of different prevention strategies, stratified by country of birth and recency of arrival. As specific countries of birth for overseas-born participants and length of residence in Australia were not collected until 2019, Fig. 3b–c and Supplemental Tables 5, 6 only show trends for 2019–2021.

HIV prevention coverage increased among Australian-born participants from 68.6% in 2017 to 73.5% in 2021 (AOR = 1.17, 95% CI 1.13–1.20, *p* < 0.001; Fig. 3a, Supp Table 4). Consistent condom use fell to a lower level among Australian-born participants than the two groups of overseas-born participants, from 28.5% in 2017 to 14.9% in 2021 (AOR = 0.84, 95% CI 0.82–087, *p* < 0.001). PrEP use and condomless sex increased among Australian-born participants from 16.0% in 2017 to 33.8% in 2021 (AOR = 1.44, 95% CI 1.40–1.48, *p* < 0.001). The proportion of Australian-born participants who were HIV-positive, undetectable and reported condomless sex was 7.0% in 2017 and 2021, but there was a downward trend in this group (AOR 0.92, 95% CI 0.88–0.97, *p* = 0.001). The was a small proportion of Australian-born participants who were HIV-positive, detectable or not on treatment, and reported condomless sex (at 0.5–0.9%; AOR = 1.07, 95% CI 0.91–1.26, *p* = 0.39). The proportion of Australian-born participants who were at higher risk of HIV infection decreased from 30.9% in 2017 to 25.6% in 2021 (AOR = 0.85, 95% CI 0.83–0.88, *p* < 0.001).

HIV prevention coverage among recently-arrived overseas-born participants remained stable between 2019 and 2021 at 72.2–74.3% (AOR = 1.19, 95% CI 0.89–1.59, *p* = 0.23; Fig. 3b, Supp Table 5). It should be noted that there was a reduced number of recently-arrived participants in the sample in 2021 (n = 81), coincident with COVID-19 travel restrictions. Recently-arrived participants were the most likely to report consistent condom use (compared with Australian-born and non-recently-arrived participants), at 32.1% in 2019 and 28.4% in 2021 (AOR = 0.89, 95% CI 0.68–1.18, *p* = 0.42). PrEP use by recently-arrived participants who reported condomless sex increased from 22.5% in 2019 to 29.6% in 2021 (AOR = 1.61, 95% CI 1.19–2.18, *p* = 0.002), but remained at a lower level than among Australian-born participants and non-recently-arrived migrants. There were relatively few participants diagnosed with HIV in the recently-arrived sample. The proportion of recently-arrived participants who were at higher risk of HIV infection was 26.9% in 2019 and 23.5% in 2021 (AOR = 0.83, 95% CI 0.62–1.11, *p* = 0.23).

Non-recently-arrived overseas-born participants (Fig. 3c, Supp Table 6) reported the highest levels of prevention coverage, increasing from 76.6% in 2019 to 79.3% in 2021 (AOR = 1.20, 95% CI 1.07–1.35, *p* = 0.002). This was associated with relatively stable proportions of participants reporting no anal intercourse and consistent condom use, and an increase in PrEP use by HIV-negative participants who had condomless sex from 32.1% in 2019 to 34.4% in 2021 (AOR = 1.13, 95% CI 1.02–1.25, *p* = 0.02). The proportion of non-recently-arrived participants who were HIV-positive, undetectable and reported condomless sex was similar to Australian-born participants, in the range 5.6–7.2% (AOR = 0.98, 95% CI 0.80–1.20, *p* = 0.86). There were very few non-recently-arrived participants who were HIV-positive, not on treatment or with a detectable viral load who reported condomless sex. The proportion of non-recently-arrived participants who were at higher risk of HIV infection declined from 22.9% in 2019 to 19.7% in 2021 (AOR = 0.83, 95% CI 0.74–0.93, p = 0.002).

### Variations in Prevention Coverage by Sexual Identity

**Fig. 4:**
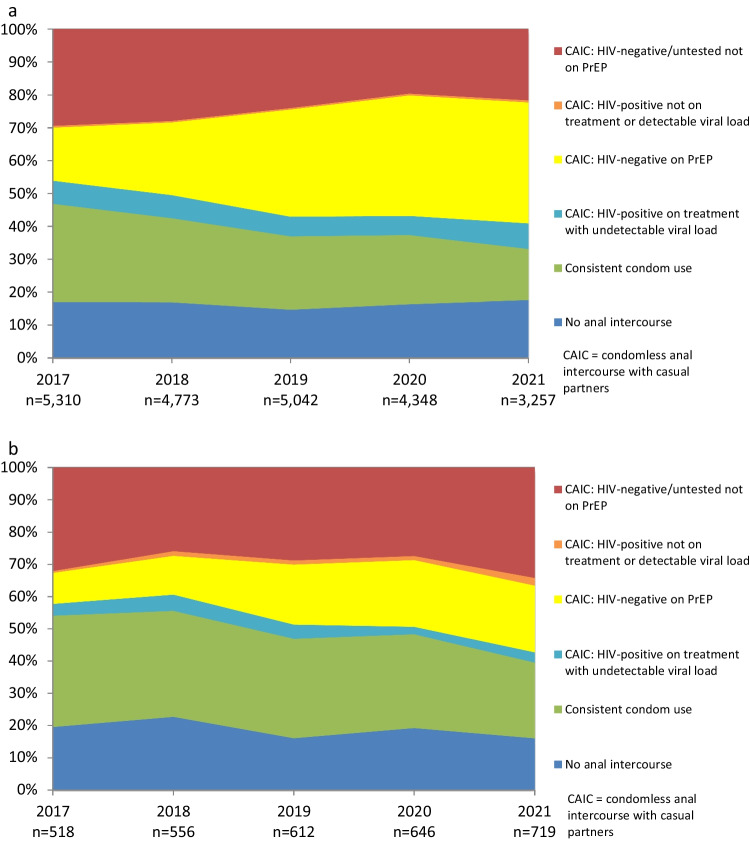
**a** Trends in the use of different prevention strategies and HIV risk with casual male partners among gay-identified participants. **b** Trends in the use of different prevention strategies and HIV risk with casual male partners among bisexual and other-identified participants

Figure 4a and b show trends in HIV prevention coverage and the use of different prevention strategies, stratified by sexual identity. Gay-identified participants (Fig. 4a, Supp Table 7) had higher levels of prevention coverage (and lower levels of casual sex with a risk of transmission) than bisexual and other-identified participants (Fig. 4b), with prevention coverage increasing from 70.1% in 2017 to 77.7% in 2021 (AOR = 1.18, 95% CI 1.16–1.21, *p* < 0.001). The proportion of gay-identified participants reporting consistent condom use fell from 29.8% in 2017 to 15.5% in 2021 (AOR = 0.84, 95% CI 0.82–0.87, *p* < 0.001). Gay-identified participants had higher levels of PrEP use during condomless sex than bisexual and other-identified participants, increasing from 16.2% in 2017 to 36.8% in 2021 (AOR = 1.43, 95% CI 1.40–1.47, *p* < 0.001). Gay-identified participants also consistently had a higher proportion of HIV-positive participants who had an undetectable viral load and reported condomless sex at 7.0% in 2017 and 7.8% in 2021, albeit with a declining trend (AOR = 0.94, 95% CI 0.90–0.98, *p* = 0.004). The proportion of gay-identified participants who were HIV-positive, detectable or not on treatment, and reported condomless sex was stable at 0.5–0.6% (AOR = 0.96, 95% CI 0.83–1.11, *p* = 0.59), while the proportion who were at higher risk of HIV infection fell from 29.4% in 2017 to 21.6% in 2021 (AOR = 0.84, 95% CI 0.82–0.86, *p* < 0.001).

HIV prevention coverage was lower among bisexual and other-identified participants (Fig. 4b, Supp Table 8) than gay-identified participants and remained stable at 67.4% in 2017 and 63.4% in 2021 (AOR = 1.05, 95% CI 0.98–1.13, *p* = 0.15). Consistent condom use was much more likely among bisexual and other-identified participants than gay-identified participants but decreased from 34.6% in 2017 to 23.5% in 2021 (AOR = 0.89, 95% CI 0.83–0.95, *p* = 0.001). The proportion of bisexual and other-identified participants who used PrEP and reported condomless sex with casual partners increased from 9.7% in 2017 to 20.7% (AOR = 1.45, 95% CI 1.32–1.60, *p* < 0.001), while the proportion who were HIV-positive, undetectable and reported condomless sex was stable at 3.7% in 2017 and 3.2% in 2021 (AOR = 0.91, 95% CI 0.76–1.08, *p* = 0.28). The number and proportion of bisexual and other-identified participants who were HIV-positive, detectable or not on treatment, and had condomless sex remained stable and low (at 0.6–2.4%). The proportion of bisexual and other-identified participants who were at higher risk of HIV infection remained stable at 32.0% in 2017 and 34.2% in 2021 (AOR = 0.95, 95% CI 0.88–1.01, *p* = 0.12).

### Variations in Prevention Coverage by Suburb

**Fig. 5:**
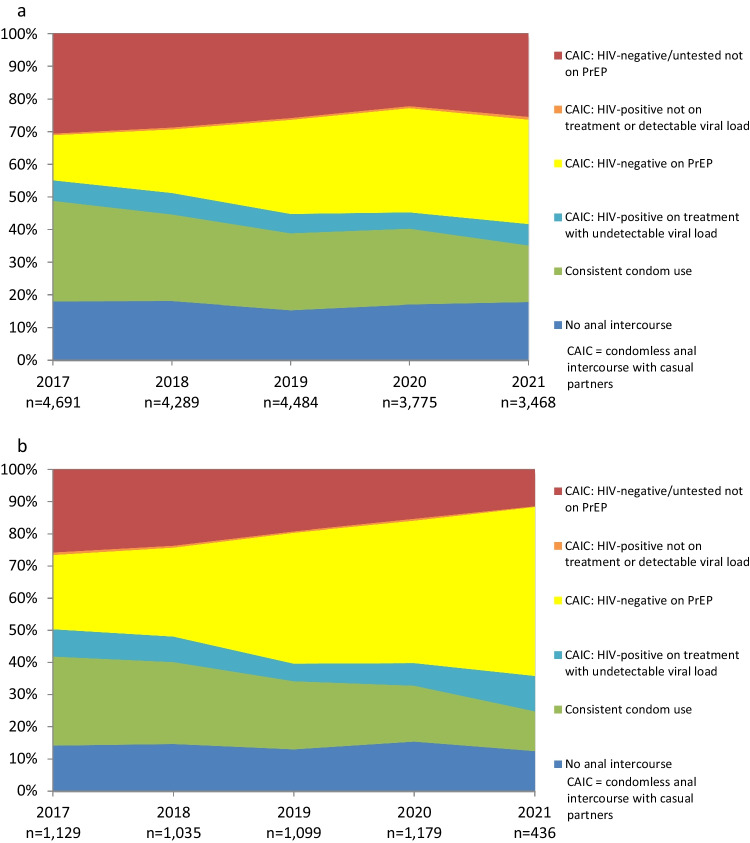
**a** Trends in the use of different prevention strategies and HIV risk with casual male partners among participants from suburbs with < 10% gay residents. **b** Trends in the use of different prevention strategies and HIV risk with casual male partners among participants from suburbs with ≥ 10% gay residents

Figure 5a and b show trends in HIV prevention coverage and the use of different prevention strategies, stratified by the proportion of gay residents in the suburb where participants live (see also Supp Tables 9–10). Most suburbs in Australia have < 10% gay residents. There are only 23 postcodes in Australia (out of over 2600) with ≥ 10% gay residents, and 17 of these postcodes with ≥ 10% gay residents are in inner city areas of Melbourne and Sydney [43]. Therefore most participants who were classified as living in suburbs with ≥ 10% gay residents were from inner Melbourne and Sydney.

Prevention coverage improved among participants from suburbs with < 10% gay residents (Fig. 5a, Supp Table 9), from 68.9% in 2017 to 73.7% in 2021 (AOR = 1.15, 95% CI 1.12–1.18, *p* < 0.001), but was consistently lower than in suburbs with ≥ 10% gay residents (Fig. 5b, Supp Table 10). The proportion reporting no anal intercourse with casual partners in suburbs with < 10% gay residents declined slightly from 18.0% in 2017 to 17.8% in 2021 (AOR = 0.97, 95% CI 0.94–1.00, *p* = 0.03). Consistent condom use remained more common in suburbs with < 10% gay residents than in suburbs with ≥ 10% gay residents, but it declined from 30.8% in 2017 to 17.3% in 2021 (AOR = 0.86, 95% CI 0.84–0.89, *p* < 0.001). PrEP use and condomless sex with casual partners by participants from suburbs with < 10% gay residents increased from 13.8% in 2017 to 32.0% in 2021 (AOR = 1.43, 95% CI 1.39–1.47, *p* < 0.001), while participants from these suburbs who were HIV-positive, undetectable and reported condomless sex was 6.3% in 2017 and 6.6% in 2021 with a declining trend (AOR = 0.94, 95% CI 0.90–0.99, *p* = 0.02). There were very relatively few HIV-positive participants in suburbs with < 10% gay residents who reported not being on treatment or a detectable viral load and condomless sex (0.5% in 2017 and 0.9% in 2021; AOR = 1.04, 95% CI 0.90–1.20, *p* = 0.58). Participants from suburbs with < 10% gay residents were consistently more likely than participants from suburbs with ≥ 10% gay residents to report sex with a higher risk of HIV infection, although this became less common over time (30.6% in 2017 to 25.4% in 2021; AOR = 0.87, 95% CI 0.85–0.89, *p* < 0.001).

Participants from suburbs with ≥ 10% gay residents (Fig. 5b, Supp Table 10) reported high and increasing levels of prevention coverage, from 73.4% in 2017 to 88.3% in 2021 (AOR = 1.26, 95% CI 1.19–1.34, *p* < 0.001). The proportion reporting no anal intercourse with casual partners in suburbs with ≥ 10% gay residents was lower than in suburbs with fewer gay residents, and was 14.1% in 2017 and 12.4% in 2021 (AOR = 1.00, 95% CI 0.94–1.07, *p* = 0.93). Consistent condom use in suburbs with ≥ 10% gay residents declined from 27.7% in 2017 to 12.4% in 2021 (AOR = 0.79, 95% CI 0.75–0.84, *p* < 0.001). Increased prevention coverage in suburbs with ≥ 10% gay residents was driven by a large increase in PrEP use by HIV-negative participants who reported condomless sex with casual partners, from 23.1% in 2017 to 52.5% in 2021 (AOR = 1.46, 95% CI 1.39–1.54, *p* < 0.001), and HIV-positive participants who were undetectable and reported condomless sex (from 8.5% to 11.0% in the same period), although this had a marginal downward trend (AOR = 0.91, 95% CI 0.84–1.00, *p* = 0.048). There were very few HIV-positive participants in suburbs with ≥ 10% gay residents who were not on treatment, or had a detectable viral load, and reported condomless sex.

The proportion of participants in suburbs with ≥ 10% gay residents who reported sex with a higher risk of HIV infection decreased from 25.8% in 2017 to 11.5% in 2021 (AOR = 0.80, 95% CI 0.75–0.85, *p* < 0.001).

## Discussion

We assessed HIV prevention coverage among Australian gay and bisexual men, and variations in coverage, HIV risk and the range of prevention strategies used by different subpopulations of GBM. During 2017–2021, we found that prevention coverage during sex with casual male partners increased (to 75%), driven by rising levels of PrEP use and high levels of viral suppression among HIV-positive men. Prevention coverage increased overall despite falling levels of condom use, but prevention coverage fell slightly between 2020 and 2021, which may be due to the disruptions associated with COVID-19 [39]. Our results build on our earlier work showing the increasing use of biomedical prevention methods and increasing prevention coverage among GBM in Australia, which have been associated with declining HIV infections [13, 17, 25]. Building on our earlier work, we identified variations in prevention coverage by subpopulation, particularly higher levels of HIV risk and lower levels of prevention coverage among younger GBM, bisexual men, and those who reside in suburbs with fewer gay residents. We also showed variation in the choice of prevention methods, with younger, recently-arrived, and bisexual GBM the most likely to use condoms, and PrEP use concentrated among gay men, 25–44-year-olds, and in suburbs with more gay residents. The use of undetectable viral load was most likely to be reported by older participants (≥ 45 years). These findings suggest opportunities to increase coverage, and some questions about the strategies promoted to different groups of GBM.

Since the introduction of PrEP, and despite COVID-19, HIV prevention coverage has continued to increase among GBM in Australia. However, at 75% in 2021 it remains far lower than the 95% target recommended by UNAIDS [11]. In 2021, the highest levels of prevention coverage we observed were among GBM aged ≥ 45 years (at 79%), non-recently-arrived migrants (79%), and in suburbs with ≥ 10% gay residents (88%). GBM aged ≥ 45 years and non-recently-arrived migrants demonstrated the most varied mix of prevention methods, employing PrEP, condoms and undetectable viral load, or avoiding anal intercourse with casual partners. The mixed pattern of prevention coverage among older GBM in particular was associated with a relatively high level of PrEP uptake, and a greater prominence of HIV-positive men and the use of undetectable viral load in this cohort [44]. In suburbs with ≥ 10% gay residents, the high level of prevention coverage was achieved by a very large increase in PrEP use (which has become the dominant strategy in those areas). Suburbs with a high proportion of gay residents are concentrated in a few inner city areas of Melbourne and Sydney, and feature a greater range of LGBTQ-friendly health services than suburban and regional areas [43]. These locations are the areas where PrEP use was most rapidly and enthusiastically embraced [45]. What would be effective to further increase coverage in groups and areas with lower levels of protection is less clear, although our results suggest some challenges and opportunities.

The lowest levels of prevention coverage we observed (and highest levels of sex with a risk of HIV transmission) were in younger GBM (< 25 years) and bisexual and other-identified participants. Coverage had been improving in both of these groups, but dropped in 2021, after COVID-19 emerged [39]. In both groups, condom use remains a more commonly used strategy than PrEP use, although (consistent with the rest of the sample) condom use has declined over time. It appears that rising levels of PrEP use in younger, bisexual and other-identified participants were interrupted after COVID-19 emerged. This suggests opportunities to increase coverage in these groups, including trying to encourage or sustain condom use for those who prefer to use them, and encouraging more GBM to consider PrEP. We acknowledge that encouraging or sustaining condom use in a context in which it is becoming much less common is likely to be challenging [46, 47], but it appears to be important if we are serious about recognising and supporting the range of prevention methods used by different strata of GBM. That said, we are not aware of research demonstrating effective ways to build confidence and skills in condom use in contexts in which PrEP and TasP are becoming normative. This would be worthy of further investigation. Encouraging PrEP use by younger, bisexual and other-identified men may be easier to achieve, if known barriers are recognised and addressed, such as providing low-cost access, publicising supportive prescribers, and increasing awareness of and use of alternative dosing options, such as on demand PrEP or long-acting injections [33, 35, 48–50]. Other barriers, such as a lack of perceived risk or concern about taking medication, may be more difficult to address [29, 48]. Highlighting potential benefits of PrEP, such as reduced anxiety about HIV and increased pleasure from sex and relationships may also be worthwhile [51–53].

We acknowledge the limitations of our analysis. Although these are the largest and longest-running surveys of GBM in Australia, they rely on repeated, cross-sectional samples mainly from metropolitan areas, and are not representative of all GBM in Australia, as bisexual men and men from regional areas are underrepresented [54]. Recently-arrived (and Asian-born) migrants may also be underrepresented in the sample, and the number of recently-arrived migrants fell in 2021, coincident with COVID-19-related travel restrictions [55]. This affected our capacity to assess trends in this group. Given the overrepresentation of recently-arrived, Asian-born GBM in recent Australian HIV diagnoses [34, 56, 57], we believe it would be useful to increase participation of this group in routine behavioural surveillance, and to assess prevention coverage and preferences for different prevention methods in this group. We believe our survey participants usually try to accurately and honestly report their sexual behaviour, but a six to twelve months recall period may be subject to recall bias, and social desirability bias may lead to under- or overreporting of sexual behaviour and HIV status [58]. Participation bias may also be present, with GBM who are more interested in HIV and sexual health being more willing to participate. Our measure of HIV prevention coverage is a conservative one, as it focuses on what participants do to prevent HIV and does not consider what their partners do. The measure may therefore inflate the level of HIV risk in the sample, i.e. HIV-negative and untested participants who are not on PrEP are classified as at risk of HIV infection if they have any condomless sex with casual partners, but their casual partners may be HIV-negative and on PrEP (PrEP sorting), or HIV-positive and undetectable (viral load sorting). When assessing trends in prevention coverage by subpopulation, we adjusted trends within each subpopulation for confounding, but we did not formally test differences between subpopulations (e.g. comparing younger and older participants). The apparent differences between subpopulations may not be statistically independent, once confounding and sample sizes have been considered.

As UNAIDS and others have noted [12, 59], there is value in assessing overall levels of HIV prevention coverage in populations like GBM, but also how the range of prevention strategies changes as newer strategies like PrEP are adopted. We have shown variations in coverage and the range of strategies used by different subpopulations of GBM. We encourage others to consider similar analyses in settings where combination prevention has been embraced. Our analysis suggests that some groups of GBM should be prioritised in order to increase the use of effective prevention methods, and assist in efforts to eliminate the sexual transmission of HIV in Australia [40, 41]. In the current analysis, younger, bisexual, and other-identified men reported the lowest levels of prevention coverage, highest levels of HIV transmission risk, were more reliant on condoms and had lower levels of PrEP use than their peers. Our analysis of subgroups with the highest levels of prevention coverage (older GBM, non-recently-arrived migrants, and suburbs with more gay residents) showed that there is more than one way to achieve high levels of coverage, such as adopting a mixture of condom use, PrEP use, and undetectable viral load, or emphasising PrEP use as the main strategy. When working to increase HIV prevention coverage in affected communities, we encourage others to support access to a range of acceptable and effective prevention strategies, rather than exclusively focusing on a single strategy.

### Supplementary Information

Below is the link to the electronic supplementary material.

Supplementary file1 (PDF 94 KB)

## Data Availability

A deidentified copy of the dataset and syntax used in these analyses is available from the authors, upon request.
