# Supplementary material for: Variations in HIV Prevention Coverage in Subpopulations of Australian Gay and Bisexual Men, 2017–2021: Implications for Reducing Inequities in the Combination Prevention Era
Source: AIDS Behav. 2023 Sep 27;28(5):1469–84. doi: 10.1007/s10461-023-04172-3 (PMC11069488; doi:10.1007/s10461-023-04172-3)
Supplement: Supplementary file 1 — Supplementary file1 (PDF 94 KB) [file 10461_2023_4172_MOESM1_ESM.pdf]

Supplemental Table 1. Trends in the use of different prevention strategies and HIV risk with casual male partners among participants aged <25 years

	2017 n (%)	2018 n (%)	2019 n (%)	2020 n (%)	2021 n (%)	AOR (95% CI)	*p* value
No anal intercourse	131 (13.7)	113 (13.9)	106 (12.7)	79 (12.8)	62 (13.4)	0.99 (0.92-1.07)	0.80
Consistent condom use	320 (33.5)	235 (28.8)	239 (28.7)	199 (32.3)	117 (25.4)	0.93 (0.88-0.98)	0.011
Any condomless anal intercourse	503 (52.7)	467 (57.3)	487 (58.5)	339 (54.9)	282 (61.2)	1.07 (1.02-1.13)	0.011
Subcategories of participants who had condomless anal intercourse							
HIV-positive on treatment with undetectable viral load	16 (1.7)	6 (0.7)	8 (1.0)	7 (1.1)	0 (0.0)	0.83 (0.62-1.10)	0.19
HIV-negative on PrEP	84 (8.8)	119 (14.6)	171 (20.6)	128 (20.7)	94 (20.4)	1.37 (1.27-1.47)	<0.001
HIV-positive not on treatment or detectable viral load	5 (0.5)	5 (0.6)	11 (1.3)	5 (0.8)	5 (1.1)	1.09 (0.81-1.48)	0.56
HIV-negative/untested not on PrEP	398 (41.7)	337 (41.3)	297 (35.7)	199 (32.3)	183 (39.7)	0.91 (0.86-1.48	<0.001
Net prevention coverage	551 (57.8)	473 (58.0)	524 (63.0)	413 (66.9)	273 (59.2)	1.10 (1.04-1.16)	0.001
Total	954	815	832	617	461		

AOR = adjusted odds ratio; CI = confidence interval

Supplemental Table 2. Trends in the use of different prevention strategies and HIV risk with casual male partners among participants aged 25-44 years

	2017 n (%)	2018 n (%)	2019 n (%)	2020 n (%)	2021 n (%)	AOR (95% CI)	*p* value
No anal intercourse	550 (15.9)	491 (16.2)	435 (13.2)	439 (14.5)	314 (15.3)	0.97 (0.93-1.00)	0.09
Consistent condom use	1,076 (31.1)	811 (26.7)	746 (22.6)	643 (21.3)	350 (17.0)	0.83 (0.80-0.85)	<0.001
Any condomless anal intercourse	1,835 (53.0)	1738 (57.2)	2126 (64.3)	1943 (64.2)	1394 (67.7)	1.18 (1.15-1.21)	<0.001
Subcategories of participants who had condomless anal intercourse							
HIV-positive on treatment with undetectable viral load	181 (5.2)	182 (6.0)	129 (3.9)	140 (4.6)	104 (5.1)	0.93 (0.88-0.99)	0.03
HIV-negative on PrEP	647 (18.7)	762 (25.1)	1,206 (36.5)	1,228 (40.6)	802 (39.0)	1.42 (1.38-1.47)	<0.001
HIV-positive not on treatment or detectable viral load	17 (0.5)	13 (0.4)	16 (0.5)	14 (0.5)	12 (0.6)	0.93 (0.77-1.12)	0.43
HIV-negative/untested not on PrEP	990 (28.6)	781 (25.7)	775 (23.4)	561 (18.5)	476 (23.1)	0.85 (0.83-0.88)	<0.001
Net prevention coverage	2,454 (70.9)	2,246 (73.9)	2,516 (76.1)	2,450 (81.0)	1,570 (76.3)	1.17 (1.14-1.21)	<0.001
Total	3,461	3,040	3,307	3,025	2,058		

AOR = adjusted odds ratio; CI = confidence interval

Supplemental Table 3. Trends in the use of different prevention strategies and HIV risk with casual male partners among participants aged ≥45 years

	2017<br>n (%)	2018<br>n (%)	2019<br>n (%)	2020<br>n (%)	2021<br>n (%)	AOR (95% CI)	*p* value
No anal intercourse	327 (22.7)	329 (22.1)	299 (19.5)	322 (23.4)	312 (21.3)	0.98 (0.93-1.03)	0.36
Consistent condom use	375 (26.1)	360 (24.2)	330 (21.6)	264 (19.2)	209 (14.3)	0.87 (0.83-0.91)	<0.001
Any condomless anal intercourse	736 (51.2)	801 (53.8)	901 (58.9)	789 (57.4)	941 (64.4)	1.11 (1.07-1.16)	<0.001
Subcategories of participants who had condomless anal intercourse							
HIV-positive on treatment with undetectable viral load	196 (13.6)	177 (11.9)	194 (12.7)	124 (9.0)	173 (11.8)	0.94 (0.89-1.00)	0.051
HIV-negative on PrEP	180 (12.5)	244 (16.4)	386 (25.2)	381 (27.7)	453 (31.0)	1.44 (1.37-1.52)	<0.001
HIV-positive not on treatment or detectable viral load	10 (0.7)	13 (0.9)	5 (0.3)	14 (1.0)	21 (1.4)	1.02 (0.81-1.29)	0.85
HIV-negative/untested not on PrEP	350 (24.3)	367 (24.6)	316 (20.7)	270 (19.6)	294 (20.1)	0.85 (0.81-0.89)	<0.001
Net prevention coverage	1,078 (75.0)	1,110 (74.5)	1,209 (79.0)	1,091 (79.3)	1,147 (78.5)	1.17 (1.12-1.22)	<0.001
Total	1,438	1,490	1,530	1,375	1,462		

AOR = adjusted odds ratio; CI = confidence interval

Supplemental Table 4. Trends in the use of different prevention strategies and HIV risk with casual male partners among participants born in Australia

	2017<br>n (%)	2018<br>n (%)	2019<br>n (%)	2020<br>n (%)	2021<br>n (%)	AOR (95% CI)	*p* value
No anal intercourse	703 (17.1)	655 (17.5)	583 (15.0)	588 (17.1)	497 (17.7)	0.98 (0.95-1.01)	0.21
Consistent condom use	1,171 (28.5)	962 (25.7)	874 (22.5)	731 (21.3)	420 (14.9)	0.84 (0.82-0.87)	<0.001
Any condomless anal intercourse	2,233 (54.4)	2,127 (56.8)	2,431 (62.5)	2,116 (61.6)	1,893 (67.4)	1.15 (1.12-1.18)	<0.001
Subcategories of participants who had condomless anal intercourse							
HIV-positive on treatment with undetectable viral load	287 (7.0)	256 (6.8)	216 (5.6)	188 (5.5)	198 (7.0)	0.92 (0.88-0.97)	0.001
HIV-negative on PrEP	657 (16.0)	793 (21.2)	1,227 (31.6)	1,190 (34.6)	950 (33.8)	1.44 (1.40-1.48)	<0.001
HIV-positive not on treatment or detectable viral load	20 (0.5)	16 (0.4)	21 (0.5)	26 (0.8)	25 (0.9)	1.07 (0.91-1.26)	0.39
HIV-negative/untested not on PrEP	1,269 (30.9)	1,062 (28.4)	967 (24.9)	712 (20.7)	720 (25.6)	0.85 (0.83-0.88)	<0.001
Net prevention coverage	2,818 (68.6)	2,666 (71.2)	2,900 (74.6)	2,697 (78.5)	2,065 (73.5)	1.17 (1.13-1.20)	<0.001
Total	4,107	3,744	3,888	3,435	2,810		

AOR = adjusted odds ratio; CI = confidence interval

Supplemental Table 5. Trends in the use of different prevention strategies and HIV risk with casual male partners among recently-arrived overseas-born participants

	2019<br>n (%)	2020<br>n (%)	2021<br>n (%)	AOR (95% CI)	*p* value
No anal intercourse	51 (15.7)	37 (13.8)	9 (11.1)	0.60 (0.48-1.00)	0.05
Consistent condom use	104 (32.1)	75 (27.9)	23 (28.4)	0.89 (0.68-1.18)	0.42
Any condomless anal intercourse	169 (52.2)	157 (58.4)	49 (60.5)	1.32 (1.02-1.72)	0.03
Subcategories of participants who had condomless anal intercourse					
HIV-positive on treatment with undetectable viral load	6 (1.9)	9 (3.3)	4 (4.9)	2.52 (0.97-6.58)	0.06
HIV-negative on PrEP	73 (22.5)	79 (29.4)	24 (29.6)	1.61 (1.19-2.18)	0.002
HIV-positive not on treatment or detectable viral load	3 (0.9)	2 (0.7)	2 (2.5)	1.16 (0.26-5.26)	0.85
HIV-negative/untested not on PrEP	87 (26.9)	67 (24.9)	19 (23.5)	0.83 (0.62-1.11)	0.22
Net prevention coverage	234 (72.2)	200 (74.3)	60 (74.1)	1.19 (0.89-1.59)	0.23
Total	324	269	81		

AOR = adjusted odds ratio; CI = confidence interval

Supplemental Table 6. Trends in the use of different prevention strategies and HIV risk with casual male partners among non-recently-arrived overseas-born participants

	2019<br>n (%)	2020<br>n (%)	2021<br>n (%)	AOR (95% CI)	*p* value
No anal intercourse	200 (14.0)	201 (15.8)	179 (16.6)	1.13 (0.99-1.30)	0.08
Consistent condom use	333 (23.4)	295 (23.1)	230 (21.3)	0.95 (0.85-1.07)	0.40
Any condomless anal intercourse	893 (62.6)	780 (61.1)	671 (62.1)	0.98 (0.88-1.08)	0.67
Subcategories of participants who had condomless anal intercourse					
HIV-positive on treatment with undetectable viral load	102 (7.2)	72 (5.6)	75 (6.9)	0.98 (0.80-1.20)	0.86
HIV-negative on PrEP	458 (32.1)	455 (35.7)	372 (34.4)	1.13 (1.02-1.25)	0.02
HIV-positive not on treatment or detectable viral load	7 (0.5)	5 (0.4)	11 (1.0)	1.05 (0.51-2.16)	0.90
HIV-negative/untested not on PrEP	326 (22.9)	248 (19.4)	213 (19.7)	0.83 (0.74-0.93)	0.002
Net prevention coverage	1093 (76.6)	1023 (80.2)	856 (79.3)	1.20 (1.07-1.35)	0.002
Total	1,426	1,276	1,080		

AOR = adjusted odds ratio; CI = confidence interval

Supplemental Table 7. Trends in the use of different prevention strategies and HIV risk with casual male partners among gay-identified participants

	2017 n (%)	2018 n (%)	2019 n (%)	2020 n (%)	2021 n (%)	AOR (95% CI)	*p* value
No anal intercourse	905 (17.0)	807 (16.9)	740 (14.7)	711 (16.4)	572 (17.6)	0.98 (0.95-1.01)	0.137
Consistent condom use	1,583 (29.8)	1,220 (25.6)	1,123 (22.3)	913 (21.0)	506 (15.5)	0.84 (0.82-0.87)	<0.001
Any condomless anal intercourse	2,822 (53.1)	2,746 (57.5)	3,179 (63.1)	2,724 (62.6)	2,179 (66.9)	1.15 (1.12-1.17)	<0.001
Subcategories of participants who had condomless anal intercourse							
HIV-positive on treatment with undetectable viral load	373 (7.0)	335 (7.0)	302 (6.0)	253 (5.8)	254 (7.8)	0.94 (0.90-0.98)	0.004
HIV-negative on PrEP	859 (16.2)	1,057 (22.1)	1,645 (32.6)	1,595 (36.7)	1,199 (36.8)	1.43 (1.40-1.47)	<0.001
HIV-positive not on treatment or detectable viral load	29 (0.5)	23 (0.5)	24 (0.5)	25 (0.6)	21 (0.6)	0.96 (0.83-1.11)	0.589
HIV-negative/untested not on PrEP	1,561 (29.4)	1,331 (27.9)	1,208 (24.0)	851 (19.6)	705 (21.6)	0.84 (0.82-0.86)	<0.001
Net prevention coverage	3,720 (70.1)	3,419 (71.6)	3,810 (75.6)	3,472 (79.9)	2,531 (77.7)	1.18 (1.16-1.21)	<0.001
Total	5,310	4,773	5,042	4,348	3,257		

AOR = adjusted odds ratio; CI = confidence interval

Supplemental Table 8. Trends in the use of different prevention strategies and HIV risk with casual male partners among bisexual and other-identified participants

	2017 n (%)	2018 n (%)	2019 n (%)	2020 n (%)	2021 n (%)	AOR (95% CI)	*p* value
No anal intercourse	101 (19.5)	126 (22.7)	98 (16.0)	124 (19.2)	115 (16.0)	0.94 (0.87-1.02)	0.16
Consistent condom use	179 (34.6)	183 (32.9)	189 (30.9)	188 (29.1)	169 (23.5)	0.89 (0.83-0.95)	0.001
Any condomless anal intercourse	238 (45.9)	247 (44.4)	325 (53.1)	334 (51.7)	435 (60.5)	1.15 (1.08-1.23)	<0.001
Subcategories of participants who had condomless anal intercourse							
HIV-positive on treatment with undetectable viral load	19 (3.7)	28 (5.0)	27 (4.4)	15 (2.3)	23 (3.2)	0.91 (0.76-1.08)	0.28
HIV-negative on PrEP	50 (9.7)	67 (12.1)	114 (18.6)	134 (20.7)	149 (20.7)	1.45 (1.32-1.60)	<0.001
HIV-positive not on treatment or detectable viral load	3 (0.6)	8 (1.4)	8 (1.3)	8 (1.2)	17 (2.4)	1.08 (0.79-1.48)	0.64
HIV-negative/untested not on PrEP	166 (32.0)	144 (25.9)	176 (28.8)	177 (27.4)	246 (34.2)	0.95 (0.88-1.01)	0.12
Net prevention coverage	349 (67.4)	404 (72.7)	428 (69.9)	461 (71.4)	456 (63.4)	1.05 (0.98-1.13)	0.15
Total	518	556	612	646	719		

AOR = adjusted odds ratio; CI = confidence interval

Supplemental Table 9. Trends in the use of different prevention strategies and HIV risk with casual male partners among participants from suburbs with <10% gay residents

	2017 n (%)	2018 n (%)	2019 n (%)	2020 n (%)	2021 n (%)	AOR (95% CI)	*p* value
No anal intercourse	843 (18.0)	775 (18.1)	686 (15.3)	642 (17.0)	616 (17.8)	0.97 (.94-1.00)	0.03
Consistent condom use	1,445 (30.8)	1,138 (26.5)	1,055 (23.5)	878 (23.3)	601 (17.3)	0.86 (0.84-0.89)	<0.001
Any condomless anal intercourse	2,403 (51.2)	2,376 (55.4)	2,743 (61.2)	2,255 (59.7)	2,251 (64.9)	1.14 (1.11-1.17)	<0.001
Subcategories of participants who had condomless anal intercourse							
HIV-positive on treatment with undetectable viral load	296 (6.3)	282 (6.6)	266 (5.9)	188 (5.0)	228 (6.6)	0.94 (0.90-0.99)	0.02
HIV-negative on PrEP	649 (13.8)	837 (19.56)	1,295 (28.9)	1,207 (32.0)	1,110 (32.0)	1.43 (1.39-1.47)	<0.001
HIV-positive not on treatment or detectable viral load	23 (0.5)	25 (0.6)	25 (0.6)	23 (0.6)	31 (0.9)	1.04 (0.90-1.20)	0.58
HIV-negative/untested not on PrEP	1,435 (30.6)	1,232 (28.7)	1,157 (25.8)	837 (22.2)	882 (25.4)	0.87 (0.85-0.89)	<0.001
Net prevention coverage	3,233 (68.9)	3,032 (70.7)	3,302 (73.6)	2,915 (77.2)	2,555 (73.7)	1.15 (1.12-1.18)	<0.001
Total	4,691	4,289	4,484	3,775	3,468		

AOR = adjusted odds ratio; CI = confidence interval; CAIC = condomless anal intercourse with casual male partners

Supplemental Table 10. Trends in the use of different prevention strategies and HIV risk with casual male partners among participants from suburbs with ≥10% gay residents

	2017 n (%)	2018 n (%)	2019 n (%)	2020 n (%)	2021 n (%)	AOR (95% CI)	*p* value
No anal intercourse	159 (14.1)	151 (14.6)	142 (12.9)	181 (15.4)	54 (12.4)	1.00 (0.94-1.07)	0.93
Consistent condom use	313 (27.7)	264 (25.5)	233 (21.2)	205 (17.4)	54 (12.4)	0.79 (0.75-0.84)	<0.001
Any condomless anal intercourse	657 (58.2)	620 (59.9)	724 (65.9)	793 (67.3)	328 (75.2)	1.19 (1.13-1.25)	<0.001
Subcategories of participants who had condomless anal intercourse							
HIV-positive on treatment with undetectable viral load	96 (8.5)	82 (7.9)	60 (5.5)	83 (7.0)	48 (11.0)	0.91 (0.84-1.00)	0.048
HIV-negative on PrEP	261 (23.1)	287 (27.7)	448 (40.8)	522 (44.3)	229 (52.5)	1.46 (1.39-1.54)	<0.001
HIV-positive not on treatment or detectable viral load	9 (0.8)	6 (0.6)	5 (0.5)	7 (0.6)	1 (0.2)	0.74 (0.54-1.01)	0.054
HIV-negative/untested not on PrEP	291 (25.8)	245 (23.7)	211 (19.2)	181 (15.4)	50 (11.5)	0.80 (0.75-0.85)	<0.001
Net prevention coverage	829 (73.4)	784 (75.7)	883 (80.3)	991 (84.1)	385 (88.3)	1.26 (1.19-1.34)	<0.001
Total	1,129	1,035	1,099	1,179	436		

AOR = adjusted odds ratio; CI = confidence interval; CAIC = condomless anal intercourse with casual male partners
